# Quintuple parasitism of a great reed warbler nest by common cuckoos

**DOI:** 10.1002/ece3.7669

**Published:** 2021-05-13

**Authors:** Attila Marton

**Affiliations:** ^1^ Department of Evolutionary Zoology and Human Biology University of Debrecen Debrecen Hungary; ^2^ Juhász‐Nagy Pál Doctoral School University of Debrecen Debrecen Hungary

**Keywords:** *Acrocephalus arundinaceus*, brood parasitism, *Cuculus canorus*, egg burial, multiple parasitism

## Abstract

Multiple parasitism in obligate avian brood parasites occurs when several brood parasitic females lay their eggs in the nest of the same host. While multiple parasitism is common in the highly social, nonevicting cowbird species (Molothrus sp.), in which multiple parasitic nestlings can be raised simultaneously by the same hosts, it is less common in the case of cuckoo species (Cuculus sp.). The first cuckoo nestling to hatch from the egg evicts all nestmates; therefore, it is costly for cuckoo females to lay eggs in already parasitized nests. However, this can occur in sites with very high parasitism rates, and it can even increase the breeding success of the brood parasites, as the presence of multiple parasitic eggs in the nest of the host decreases rejection rates. Here, we present a case of a quintuple brood parasitism of a great reed warbler (*Acrocephalus arundinaceus*) nest, an extreme form of multiple brood parasitism.

## Graphical abstract

Multiple parasitism of a host nest by several females is rare in the case of the common cuckoo (*Cuculus canorus*), an obligate brood parasite. However, this can occur in sites with very high parasitism rates, and it can even increase the breeding success of the brood parasites, as the presence of multiple parasitic eggs in the nest of the host decreases rejection rates. Here, we present a case of a quintuple brood parasitism of a great reed warbler (*Acrocephalus arundinaceus*) nest, an extreme form of multiple brood parasitism.

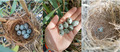

## 1 INTRODUCTION

Obligatory brood parasitism represents a rare avian breeding strategy, characteristic to only 1% of the bird species existing today (Davies, 2015). These 102 brood parasitic species inhabit all continents except Antarctica, and breed by laying their eggs in the nests of other birds and by tricking these host species into raising the parasitic nestlings (Payne, 2005). Once hatched, the brood parasitic chicks greatly reduce the breeding success of the hosts, by outcompeting the hosts’ offspring or by killing their nestmates altogether (Davies, 2000). Therefore, hosts have evolved multifaceted defenses to avoid being parasitized (Feeney et al., 2012; Welbergen & Davies, 2009), such as building highly concealed nests (Jelínek et al., 2014; Mérő & Žuljević, 2015, 2019), gathering social information regarding the presence of brood parasites (Lawson et al., 2020, 2021; Thorogood & Davies, 2012, 2016), and by mobbing adult brood parasites (Požgayová et al., 2009; Trnka et al., 2013). Brood parasites bypass these defenses by fast egg‐laying (Jelínek et al., 2021), color dimorphism (Thorogood & Davies, 2012), visual and acoustic mimicry (Marton et al., 2021; Trnka & Prokop, 2012; Welbergen & Davies, 2008; York & Davies, 2017), and sometimes also by retaliatory strikes and brood destruction (Soler et al., 1995). If brood parasites find and parasitize a host nest, the host aims to minimize the negative effect of the successful parasitic event, if recognized in time, by abandoning the nest, burying the parasitic egg inside the nest lining, or removing the egg (Molnár, 1944; Moskát & Honza, 2000) or nestlings (Langmore et al., 2003) from the nest.

In order to maximize their breeding success, obligate brood parasitic females lay only one egg into every host nest, although some nests may be found and parasitized by multiple females (Davies, 2015; Gloag et al., 2014). In such cases, the first laid brood parasitic egg hatches earlier then the subsequently laid parasitic eggs, which reduces the reproductive output of these late‐arriving females. This strong selective pressure should select for females that parasitize host nests as soon as the hosts’ start laying eggs, or for parasitic females which are able to recognize and remove the previously laid eggs of conspecifics. Intriguingly, previous studies found that brown‐headed cowbirds (*Molothrus ater*) and common cuckoos (*Cuculus canorus*), the two most widely studied brood parasites, are unable to differentiate between their hosts’ eggs and the eggs previously laid by another brood parasitic female (Goguen et al., 2011; Šulc et al., 2016; Yang et al., 2016, 2017).

The parasitism of host nests by multiple brood parasitic females is known to be fairly common in cowbird species (Molothrus sp., Ellison et al., 2006; Hoover, 2003; McLaren et al., 2003; Tuero et al., 2007) and in the greater spotted cuckoo (*Clamator glandarius*, Soler & Soler, 1999). Per example, in a study conducted by Goguen et al. (2011), of the 642 nests of three host species examined during a 11‐year period, 74% of were parasitized by cowbirds, of which 38.2% were found to be parasitized by multiple cowbird females, but only 15 nests received >3 cowbird eggs. On the other hand, the hosts of the common cuckoo generally face a parasitism rate of 10%–35%, rendering the occurrence of multiple parasitism as unlikely (Antonov et al., 2006, 2007; Avilés et al., 2005; Stokke et al., 2007; Rutila et al., 2002; Yang et al., 2014).

Our study site on the Great Hungarian Plain stands out as a striking exception regarding the parasitism rate: Here, the great reed warbler (*Acrocephalus arundinaceus*) hosts breed in narrow reed beds along irrigation channels and face a parasitism rate of 50%–60% and a multiple parasitism rate of 10%–25% (Moskát & Honza, 2000; Zölei et al., 2015). Data from locally GPS‐tagged common cuckoos show that both female and male common cuckoos actively hold ca. 2 km long overlapping territories along the irrigation channels, although some individuals may cover larger distances in order to find suitable host nests (Moskát et al., 2019). This locally high density of cuckoos probably contributes to the high abundance of multiple parasitized nests, since cuckoos eavesdrop on the alarm calls of their hosts, and multiple females might cue in on the mobbing calls of their hosts (Marton et al., 2019; Moskát et al., 2017).

## 2 RESULTS

**FIGURE 1:**
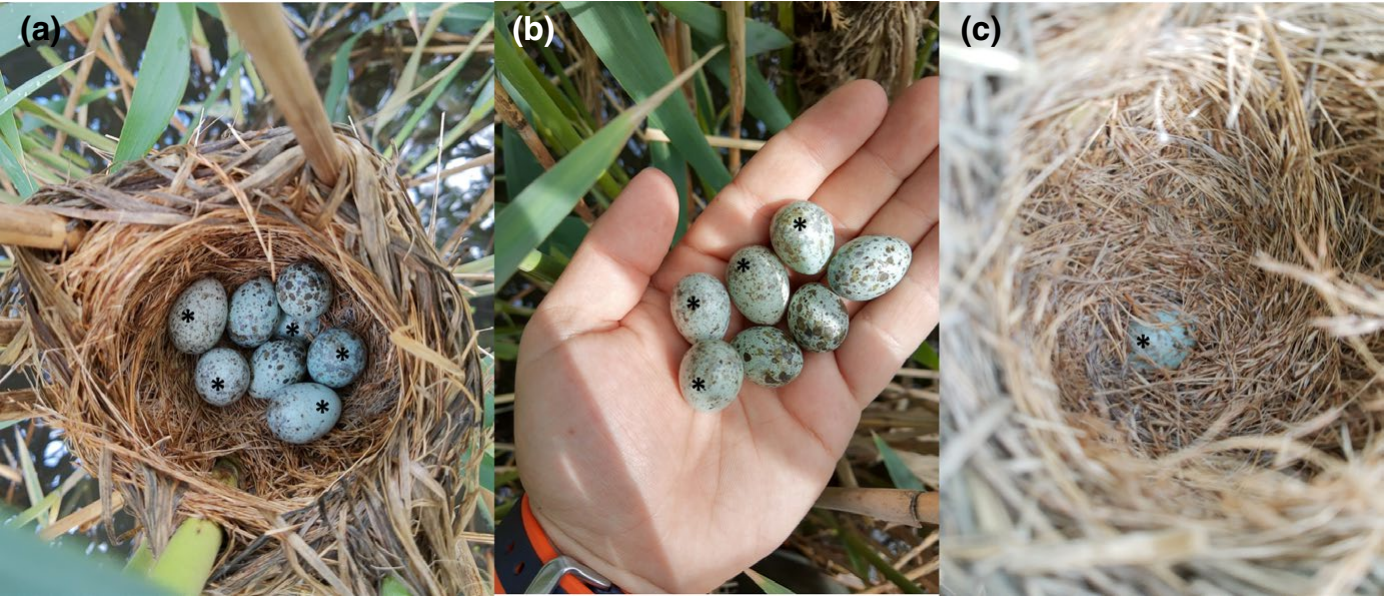
Quintuple parasitism by common cuckoo in a great reed warbler nest with three host eggs (a), three host eggs and four parasitic eggs in hand (b), and one parasitic egg buried in the bottom of the nest (c). All common cuckoo eggs are marked with an asterisk

On the 16th of May 2020 during our regular great reed warbler nest searching and nest checking activity at our study site near Apaj village, central Hungary (47.113°N, 19.087°E), we found a nest containing three great reed warbler eggs and five common cuckoo eggs, with one of the cuckoo eggs being buried in the bottom of the nest (Figure 1). Although the nest was at a distance of ca. 1.5 m and directly visible from a fishing stand often used by local fishermen, one of the common cuckoo nestlings hatched from the egg on the 26th of May and ejected the remaining eggs from the nest, except the one buried in the nest lining. The nest was subsequently checked only once, on the 6th of June, when we found the common cuckoo nestling alive and in a good condition.

## 3 DISCUSSION

While the parasitism of the same host nest by several female brood parasites is well known in cowbirds, multiple parasitism is less common in the common cuckoo. Here, we present an extreme case of multiple parasitism by the common cuckoo, where five parasitic eggs were laid most probably by five different common cuckoo females in the nest of a great reed warbler.

Our knowledge regarding the occurrence of multiple parasitism by cuckoos (Cuculus sp.) in nature might be biased because, due to logistic reasons, the relationship between these brood parasites and their hosts is generally studied at sites with high parasitism rates (Antonov et al., 2006, 2007; Avilés et al., 2005; Stokke et al., 2007; Rutila et al., 2002; Yang et al., 2014; Zölei et al., 2015). Nevertheless, multiple parasitism by more than two females is still only rarely recorded even in these sites with high parasitism rates (Yang et al., 2014; Zölei et al., 2015). Great reed warblers breeding at our study face a parasitism rate by common cuckoos of 50%–60%, which is one of the highest rates ever recorded for this brood parasite worldwide. Here, triple parasitism occurs at a rate of 3%–6% occurs, while quadruple parasitism occurs at a rate of <2% (Zölei et al., 2015). To the best of our knowledge, the only other case of quintuple parasitism was recorded by Molnár (1944), in the nest of the same host species, in a similar habitat with irrigation channels.

The narrow reed beds along the irrigation channels run parallelly to lines of tall trees from where cuckoos can eavesdrop on hosts (Marton et al., 2019), and are the preferred breeding sites of great reed warblers (Báldi, 1999; Báldi & Kisbenedek, 1999). Early arriving, high‐quality great reed warbler males occupy these habitats prefer these “edge‐habitats” at the detriment of their own breeding success, as previous studies have shown that these irrigation channels have high parasitism rates and act as ecological traps for the hosts (Mérő, et al., 2015; Mérő et al., 2015, 2020). Therefore, it is likely that this conjuncture of high nesting host density, a high probability for the parasite to find host nests in the narrow reed bed, high density of parasites, and the occasional improper management of the reed (Mérő, Lontay, et al., 2015; Mérő, Žuljević, et al., 2015; Mérő et al., 2018) can facilitate the appearance of multiple brood parasitism.

Due to the longstanding high local parasitism rate, it would have been expected that, under strong selective pressure, great reed warblers would recognize multiple parasitic eggs and remove these eggs from their nests. However, previous studies conducted locally have shown that multiple parasitism, instead of functioning as a cue that the nest was parasitized, reduces egg rejection rates by the host (Manna et al., 2019; Moskát et al., 2009). These results show that great reed warblers reject foreign eggs based on their discordance to the rest of the eggs within the nest, rather than truly recognizing their own eggs, and multiple parasitism can even double the breeding success of common cuckoos per host nest by increasing the tolerance of the hosts toward dissimilar looking eggs (Moskát et al., 2009). Therefore, future studies are warranted in addressing the questions regarding the costs and benefits of multiple parasitism for nestmate‐evicting brood parasites, and the publication of brood parasitism rates on a wider geographic rage should also be encouraged.

## CONFLICT OF INTEREST

None declared.

## AUTHOR CONTRIBUTIONS

**Attila Marton:** Investigation (lead); project administration (lead); visualization (lead); writing–original draft (lead); writing–review and editing (lead).

## Data Availability

Data sharing is not applicable to this article as no new data were created or analyzed in this study.
